# A Social-Ecological Approach to Identify Facilitators and Barriers of Home Modifications

**DOI:** 10.3390/ijerph18168720

**Published:** 2021-08-18

**Authors:** Carlyn Ellison, Linda Struckmeyer, Mahshad Kazem-Zadeh, Nichole Campbell, Sherry Ahrentzen, Sherrilene Classen

**Affiliations:** 1 Department of Occupational Therapy, University of Florida, Gainesville, FL 32611, USA; lstruckmeyer@phhp.ufl.edu (L.S.); sclassen@phhp.ufl.edu (S.C.); 2 Rinker School of Construction Management, College of Design, Construction and Planning, University of Florida, Gainesville, FL 32611, USA; m.kazemzadeh@ufl.edu; 3 Shimberg Center for Housing Studies, University of Florida, Gainesville, FL 32611, USA; ahrentzen@ufl.edu; 4 Interior Architecture, Ohio University, Athens, OH 45701, USA; ncampbell@ohio.edu

**Keywords:** accessible interior design, architectural accessibility, environment, independent living, public health

## Abstract

Aging individuals may face difficulty with independently navigating and interacting with their home environment. Evidence-based interventions promoting home modifications are needed to support aging-in-place across the lifespan. This study identified the facilitators and barriers to implementing home modifications from the perspectives of residents and professionals (*N* = 16). Guided by a social-ecological model, researchers utilized directed content analysis of focus group interviews. While participants discussed facilitators and barriers mainly on the individual level, factors were presented at the relationship, community, and societal level of the model. Overall, the findings suggest a potential for targeted interventions on all levels of the model to promote adoption of home modifications.

## 1. Introduction

The Centers for Disease Control and Prevention (CDC) defines aging-in-place as “the ability to live in one’s own home and community safely, independently, and comfortably, regardless of age, income, or ability level” [1]. Harrell et al. found that 87% of those aged 65 years and greater indicated a desire to remain in their current home as they age [2]. Commonly, individuals view aging-in-place as desirable because staying in their existing home allows them to remain independent and comfortable, as well as being the most cost-effective solution for many [3]. As such, aging-in-place is a component of active aging, as the World Health Organization (WHO) recommends “creating physical and social environments that foster the health and participation of older people” [4]. However, an increase in functional limitations poses challenges to an aging individual’s independence when navigating and interacting within their homes. This present study identifies the facilitators and barriers to implementing home modifications from the perspectives of both residents and professionals.

The current housing stock contains more inaccessible than accessible homes. Less than 5% of U.S. homes have accessible features for individuals experiencing moderate mobility difficulties [5]. Thus, individuals needing accommodations rely on retroactive home modifications to enhance the environment for their accessibility needs.

Home modifications can address accessibility needs, improve the quality of life, and promote independence [6]. Yet, despite their utility, residents may not implement home modifications due to cost and aesthetics [7]. The purpose of this study is to further identify facilitators and/or barriers for home modifications through the perspectives of residents and professionals. Utilizing a social-ecological model, the researchers aim to identify facilitators and barriers beyond the individual level, which will illuminate areas where interventions could promote home modifications for supporting aging-in-place.

### 1.1. Home Modifications and Aging-in-Place

Home modifications can range from minor changes, such as removing rugs that pose a fall risk, to extensive renovations of the home, such as remodeling an entire bathroom. These modifications can decrease the cost burden of healthcare for aging individuals by helping to manage the functional limitations associated with chronic health conditions [8,9] and prevent falls [10]. Home modifications may also potentially prolong independence at home, or in some cases, delay institutionalization of older adults [11]. Additionally, people living with a disability at any age may benefit from home modifications [12]. Thus, home modifications may act as a bridge to emphasize active aging through a life course perspective [4].

An ecological theory of aging indicates that a match must exist between an individual’s abilities and their environment to adequately participate in required and desired daily activities in the home [13]. Researchers have identified barriers in the home environment that influence the implementation and initiation of home modifications [14,15]. However, the barriers are primarily at the individual level. Therefore, a specific inquiry is needed to understand the individual, relationship, community, and environmental factors supporting or inhibiting individuals’ plans to implement home modifications.

### 1.2. Social-Ecological Model

A social-ecological model reveals the facilitating and inhibiting factors associated with human behaviors. In this study, the model guided our explorations of the factors related to implementing and using home modifications. The model was also used to help identify factors “beyond the influence of the health system” that may be a determinant of active aging [16]. The CDC developed a social-ecological model that served as the conceptual framework for this study [17]. The model contains four levels: individual, relationship, community, and societal. Individual-level factors pertain to the knowledge, attitudes, and beliefs of an individual (e.g., aesthetic preferences). Relationship-level factors refer to the influence of individuals within their social network (e.g., peers and family) that may influence decisions on home modifications. Community-level factors include the various settings in which participants live and work (e.g., Homeowners’ Associations (HOAs) that may affect their ability to implement home modifications). Finally, policy-level factors focus on the national, state, and local laws that influence an individual’s behavior (e.g., zoning laws that may impact how quickly a renovation can occur). Therefore, the social-ecological model may point to factors previously unaddressed in the literature pertaining to home modifications.

Approaches that utilize a social-ecological framework to target multiple-level factors—rather than a single level, such as aesthetic preferences at the individual level—can impact health behavior [18]. While health professionals may educate individuals about the importance of home modifications that impact health, education alone may not be sufficient to result in sustainable and safe changes within the home. Likewise, community groups (e.g., Rebuilding Together [19]) might provide resources for implementing home modifications, but this does not guarantee that people will utilize the available resources. For example, individuals may not have a way to access the community resources due to lack of transportation, and funding may be limited. As such, the interaction of the various factors at each level of the model can have a cumulative effect on promoting or hindering the desired behavior [18]. Therefore, this study uses the CDC’s social-ecological model [17] to identify various level factors that may act as facilitators and barriers to home modifications. As a result, the study will help uncover facilitators and barriers beyond the individual level.

## 2. Methods

### 2.1. Compliance with Ethical Standards

The University of Florida’s Institutional Review Board-02 approved this study. The researchers obtained informed consent from each participant. The research team maintained the confidentiality, privacy, and security of the participants’ information according to university protocol. There were no personal conflicts of interest involved in this study, and the participants received a $25 gift card after completing the focus group interview.

### 2.2. Design

The researchers employed a qualitative research design to scrutinize data collected from two focus groups from a larger study (i.e., Project Re-Envision [20]).

### 2.3. Setting

At a local Center for Independent Living, focus group sessions took place in a private room intended to optimize comfort, convenience, and privacy for participants. The facility complied with the American Disability Act (ADA) guidelines, and as such, was accessible for participants with functional mobility limitations and/or visual impairments. 

### 2.4. Participant Recruitment

The researchers of the parent study [7] used purposive sampling to target participants based on the study objectives [21], and snowball sampling was encouraged. As such, the study’s advisory board, study team members, and local professionals working in related fields recommended participants. The researchers provided marketing flyers to potential participants. To enroll participants, researchers contacted the prospective participants via phone call or email using the university’s secure email server. The researchers continued to recruit until the maximum number of participants was reached. The following section will describe the two types of participants, residents and professionals, and their inclusion and exclusion criteria.

#### 2.4.1. Residents

Residents were defined as individuals who could benefit from or have had home modifications. More specifically, participants had low vision or functional mobility limitations.

##### Inclusion and Exclusion Criteria

Residents recruited for the focus groups were required to be 18 years of age or older. More specifically, participants included in the resident group must have reported low vision or functional mobility limitations. The parent study defined a self-report of low vision as having impaired, yet functional, travel vision [7]. Participants were excluded if they were unable to be physically present at the site of the focus group interview or individuals without fluency in English.

#### 2.4.2. Professionals

Professionals were defined as individuals 18 years of age or older with experience devising or recommending home modifications. Participants of the professional group included individuals recruited from professions involved in the home modifications process, such as design, building, or healthcare professions.

##### Inclusion and Exclusion Criteria

Professionals recruited for the focus groups were required to indicate that they had experience devising or recommending home modification solutions to assist residents with functional limitations. Again, the exclusion criteria included individuals unable to attend the focus group interviews in person or individuals without fluency in English.

### 2.5. Protocol

Each of the two 90-min in-person focus group sessions had a moderator and three other research personnel present. After the moderator described the study aims, the research team members obtained informed consent and administered a brief demographic survey. The moderated focus group discussion mainly centered around which needed home modifications were the most difficult to resolve. Moreover, the discussion also involved sharing stories of innovative solutions for challenges within the home, as described in the parent study [7].

### 2.6. Data Management

The transcripts, previously verified for accuracy with audio recordings and de-identified, were stored in Word documents maintained within password-protected computers. The researchers uploaded the original transcripts into a new project within NVivo 12 software (QSR International Pty Ltd., Melbourne, Australia, Version 12, 2018). Additionally, the researchers utilized all the data from the original transcripts for analysis, which is described further in the following section.

### 2.7. Data Analysis

The researchers employed the directed content data analysis approach to code the influences described by the participants. The coding process was informed by the CDC’s social-ecological model [17]. The researchers completed the first round of coding, and a corresponding codebook was developed. Subsequently, and based on the developed codebook, two additional research team members independently coded the transcripts. The research team then conducted a group review in which discrepancies were resolved via consensus. Next, the researchers conducted a second cycle of coding to group codes into salient themes (henceforth referred to as factors). The factors were then vetted for accuracy by a qualitative analysis colloquium group affiliated with the university.

## 3. Results

### 3.1. Participant Characteristics

Sixteen participants completed the focus group interviews, including a resident group (*n* = 8) and a professional group (*n* = 8). The majority of the resident group (62.5%) owned their home, one participant indicated they were currently renting, and two participants chose not to answer. This group also included residents with various challenges as 62.5% indicated they used a form of mobility-assistance equipment within their home (e.g., wheelchairs or canes). Additionally, half of the participants indicated they used some type of adaptive equipment to assist with vision at home (e.g., magnifiers).

The professionals represented occupational therapists (*n* = 3) and a physical therapist, as well as housing professionals, including a building consultant, an architect, a nonprofit home renovator, and a chief financial officer for a developer/builder. Additionally, a wide range of years in their respective professions was represented, including 25% with five or fewer years, 37.5% with six to 20 years, and 37.5% with greater than 20 years.

### 3.2. Individual

**Figure 1:**
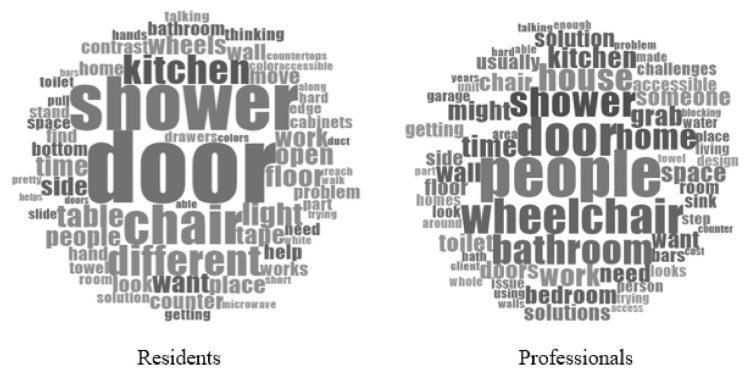
Word clouds of frequent words discussed by residents and professionals.

At the individual level (i.e., knowledge, attitudes, and beliefs), two specific factors emerged from the data, which included: needs/preferences and affordability. Specifically, an individual’s needs and preferences, as well as affordability, influenced the individual’s choice in implementing home modifications. Individual factors were discussed with the highest frequency (*n* = 48 times by residents; *n* = 50 times by professionals) by all participants (see Table 1). Further, frequency count word clouds from the transcripts compare the most discussed words from each group (see Figure 1).

#### 3.2.1. Needs and Preferences

The first factor of the individual level, needs and preferences, focused on residents’ individual needs and preferences due to their disability or aesthetic inclinations. For example, a resident with visual impairments shared that when “countertops are too busy, it’s hard to find things.”

One professional emphasized that the home environment is not all that needs to be evaluated:

*“… To really not just evaluate the space but how they move and operate within that space and which areas they spend the most time in or what they do the most in those areas. And then time for decision making and talking together about what they would consider doable, or they would even consider, you know…in this menu of options you have, which part of the menu are you agreeable to even consider?*”

The participant recognized that the home modification process involved more than just the environment itself. In her own words, she explained, “… I’m coming in with my own … perspective on what’s safe or not safe, and for the residents, they often think … that’s not something to worry about, we’ll just skip over that.” Her explanation provides evidence that individual needs and preferences must be considered in the home modification process, which was echoed by other professionals. Therefore, individuals need home modifications that support their needs and preferences to have buy-in for changes to their homes.

#### 3.2.2. Affordability

The participants frequently discussed the affordability of home modifications and how lack of affordability was perceived as a barrier. Residents discussed other strategies that could be implemented to avoid any costs while still accommodating their needs. For example, one participant discussed how low vision challenged her ability to get food out of the microwave that is located above her height (i.e., fixed above the stove) difficult:

*“I … remember like how big the plate is, and I try to go as back as far as I can before I set it down, and then I … use like my sense of touch as a gauge to kind of maneuver it.”*

The resident also discussed how she had memorized her bathroom by rug placement as a strategy to avoid high-cost remodeling. She associated the placement of colored rugs with areas in the bathroom to help orient herself when navigating the space. Overall, it was common for participants to share adaptations to behavior or environment that required little to no cost, as affordability of the home modifications greatly impacted implementation.

A professional explained, “It’s mostly low-income people in [my area of practice], and it’s just, the remodel isn’t even a consideration, we wouldn’t even bring it up. I mean, it would be hurtful to bring it up honestly.” In his description, the participant showed how even discussing home modifications with individuals can be difficult when considering their income.

On the other hand, one resident explained a more optimistic view of the cost of home modifications:

*“Even from then [1990s] to now this stuff that was really, really cost-prohibitive and expensive … [but] these ideas we are coming up with they might be cost-prohibitive now but next week they won’t be. Somebody, some entrepreneur will come up and say, ‘okay, this need, I can make a bunch of money if I do this … and so I’ll get a quarter, I’ll sell millions of them.’”*

The participant seemed to hope that home modifications will slowly go down in price as new technology is developed and mass usage increased. Overall, the affordability of home modifications for residents played a large role in the adoption of home modification recommendations. As demonstrated in this study, affordability was considered a barrier due to the actual financial constraints of the individuals needing the modifications.

### 3.3. Relationship

Both groups expressed statements about their relationship with each other (i.e., residents and professionals’ relationships) as well as relationships within their network. The three factors discussed include the relationships among residents and family/caregivers, residents and professionals, and among professionals, next described in detail.

#### 3.3.1. Resident and Family/Caregivers

Caregivers and family members often provide support to those who need home modifications. One occupational therapist explained, “There’s not a lot to do unless there’s a caregiver or someone who’s willing to be a part of the solution.” This participant, along with others, emphasized the importance of supportive relationships in the home modification process.

Residents shared how relationships can hinder their ability to implement home modifications. For example, one resident with low vision shared that she “moved back to [her] childhood home and [her] mom actually chose those countertops. So eventually [her mom] says she is going to change them into granite which is like kind of nice cause it’s not so busy.” Since she has poor vision, the resident prefers countertops that are uniform in color to provide contrast between the countertops and items placed on the counter’s surface. Her mother chose the countertops without considering the needs or preferences of her daughter. This suggests families and caregivers can influence the need for home modifications and can opt to do so in a positive way by being “willing to be a part of the solution,” as one occupational therapist described.

Further, family members and residents may have differing opinions regarding values and concerns, which can hinder the home modification process. For example, when discussing privacy, an occupational therapist shared, “When you are working with caregivers and a client at the same time, the client might not like that [a toilet positioned at the front of a bathroom door], but the caregiver is thrilled that they [the caregivers] have a visual line of sight.” In this way, the caregiver would be able to easily see the resident and provide assistance, if necessary, but the resident may value their privacy more. The occupational therapist added, “I think there’s compromise on those [disagreements],” which highlights how important working with both the resident and their family/caregivers is for making solutions in the home.

#### 3.3.2. Resident and Professional

Participants in both focus groups shared aspects of the resident and professional relationship that may impact individuals implementing home modifications. One occupational therapist stated how it is important to have time to work with both the resident and their family to determine their needs within their space. Additionally, a resident with limited functional mobility focused on how it may be difficult “finding a builder or an architect that will make [a rotating handle solution for transfers in the bathroom].” As relationships are unique, their influence may act as both barriers and facilitators in the home modification process, as identified in this study.

#### 3.3.3. Professional and Professional

Another factor at the relationship level emerged from the professional group and their relationships with other professionals involved in the home modification process. A housing consultant expressed their frustration specifically regarding builders and the tendency for standard building practices not to include adaptable home features. She stated that she wanted builders to “just take the time to put blocking in the bathroom and also three-foot [wide] doors, how much more does it cost?” Additionally, a chief financial officer for a developer/builder shared that the “misadventure with home modifications is getting the help that is competent and doing it easily.” In other words, they shared that it can be challenging to find individuals who perform adequate home modifications. As anticipated by the social-ecological model, this study shows that each unique relationship (i.e., resident and family/caregivers, resident and professional, and professional and professional) may act as a facilitator or barrier to the implementation of home modifications.

### 3.4. Community

The community also influences a resident’s decision to adopt home modifications. Factors that influence the implementation of home modifications at the community level included community resources and neighbors, more specifically, support or lack of support from organizations and neighbors. The following section will describe the two sub-themes, community resources and neighbors, in detail.

#### 3.4.1. Community Resources

The residents shared the names of community resources with each other at various times throughout the session, which indicates the importance of awareness regarding resource options available to residents. As one occupational therapist stated, “… I guess it’s kind of old school, but a lot of patients end up using their churches or, you know, community organizations …” Indicating that this implementation strategy is “old school,” may reinforce the idea that utilizing a church community for support may be a long-held custom that is an integral way for many individuals to gain access to home modifications. Some other community resources mentioned included nationally known resources such as the Center for Independent Living, Veterans Affairs Medical Centers, and Meals on Wheels. In addition, participants discussed local community resources, which included resource centers specific to the town where the research was conducted. One participant also shared their experience regarding the benefits of using the Internet as a community resource. As such, the availability and awareness of community resources may contribute to individuals implementing home modifications.

The same occupational therapist shared that, “It’s kind of like knowing the right people at the right time. Kind of like the luck of the draw, but a lot of people end up utilizing religious organizations.” In this sense, the participant demonstrates the pitfalls of being reliant on community resources because needed resources may or may not be available when an individual requires them. Therefore, community resources may be essential for home modifications. Still, options for home modifications may be limited due to the financial constraints of residents, awareness of potential resources in their community, and access to community resources (including the availability of skilled trade labor). Therefore, these challenges hold a plausible opportunity for expanding funds for community organizations to provide educational and monetary resources for individuals seeking home modifications.

#### 3.4.2. Neighbors

Professionals, but not residents, minimally discussed the impact of neighbors on the implementation of home modifications. Regarding the practice of pooling resources among neighbors, one occupational therapist shared, “These are really low-income people, the grab bar might come from their neighbor you know whose husband died, I mean it’s a really backdoor deal, there’s no money.” This statement underscores how differing levels of the model can influence one another (e.g., the influence of community on the individual level), and this example highlights how neighbors can facilitate the home modification process by offering their available surplus resources.

Another view of neighbors showed that they may not always be facilitators of home modifications. A housing consultant described “neighborhood resistance” as follows:

*“[Home modifications] may even have a local government blessing. You can do this in certain neighborhoods, but the neighbors themselves don’t want it ‘cause they think it’s gonna take down the value of their property to do that.”*

In other words, home modifications (e.g., entry ramps into the home and granny flats) that others can see can cause neighbors to become a barrier because these types of solutions may impact the value of neighboring properties. Taken together, residents and professionals in the study pointed to community-level factors as integral for residents to implement home modifications. The participants’ perspectives suggest that factors at the community level can act as facilitators of the home modification process if resources are available, known, and accessible. However, neighbors can act as both a barrier and facilitator to the home modification process.

### 3.5. Societal

At the societal level (e.g., influences of policy and culture), participants shared factors that more broadly influenced the home modifications process for residents. Professionals commented 17 times on this topic, compared to one time by residents. The professional group’s responses focused on laws and social norms. Therefore, professionals were more likely to share societal-level factors when compared to the responses of the resident group.

#### 3.5.1. Laws

Laws that can impact the implementation of home modifications can range from local to national. A chief financial officer for a developer/builder shared two instances where the law can be an issue. He explained that “zoning hearings can take months if not years.” As a result, individuals may be forced to live in environments that are not accommodating their needs for an extended period. He also shared his opinion on the ADA standards stating that, “ADA is one size that fits all, and it doesn’t really work.” As this sentiment was later echoed throughout the discussion, this suggests that overarching laws still fall short of meeting the individualized needs of residents. As such, professional participants in the study identified the need for laws that are inclusive of the many factors that impact health behavior and accommodate the varying needs of residents.

#### 3.5.2. Social Norms

Social norms may influence what laws and behaviors are commonplace in a culture. Participants primarily focused on the norms of the residential building industry rather than the culture of residents. A housing consultant highlighted the issue with the present way we expect individuals to be responsible for making their home accessible for aging-in-place:

*“Is there a cheaper way to do it just do it from the get-go? Make the homes universal design or at least … a bedroom and a bathroom on the first floor that you can… that has the three-foot door to make it accessible.”*

Universal design principles exist, yet they are not deployed in a fully systematic or consistent way. A resident with functional mobility limitations stated, “Builders in the first place have a mindset that the bathroom door doesn’t need to be any wider than they are.” The participants’ comments suggest a need exists to create awareness among builders for the demand for universal design features.

In another example, an architect exemplified the impact of social norms:

*“I was just thinking that almost all solutions are usually based on some short-term thought process … we just need to accommodate somebody for six months, a year, two years … it would be wonderful if houses came a little better prepared for a longer-term solution instead of having to be modified for a short-term solution, but I don’t know how you get there.”*

The architect’s statement emphasizes how the current social norms regarding housing emphasize short-term solutions rather than long-term planning. Following his statement, another participant, a housing consultant, replied with one word: “advocacy.” This illustrates how social change could be a positive influence in the home modification process. However, the subject was not further discussed by participants. Consequently, this study identifies that, in a professional’s opinion, factors within the societal level, including laws and social norms, can promote or hinder the implementation of home modifications.

## 4. Discussion

Using a conceptual model to organize the participant opinions, this secondary analysis identified the facilitators and barriers of home modifications by residents and professionals via focus group interviews. The researchers aimed to expand the scope of facilitators and barriers to illuminate areas where home modification interventions may promote aging-in-place. Due to the variety of factors that may influence home modifications, the researchers grouped factors by levels according to a social-ecological model [17]. Focus group data provided evidence that there are factors at all levels of the social-ecological model that influence the implementation of home modifications.

### 4.1. Individual Level

Specifically, for the individual level, the researchers confirmed that needs and preferences may influence a resident’s desire for home modifications. Similarly, other studies indicated resident-centered needs and preferences [22,23,24]. Consistent with Lee et al., this study provides individualized solutions [3]. Specifically, professionals may help their residents to adopt home modifications more easily by focusing on the affordability of options. Lee and colleagues’ work also emphasized the importance of low-cost home modifications [3]. The researchers conducted 30 interviews with low-income, elderly homeowners, and many of the participants who implemented home modifications also reported better housing conditions and satisfaction after the repairs.

### 4.2. Relationship Level

At the relationship level, the participants shared the importance of the role of caregivers and family for a person with functional mobility and visual limitations. These intimate relationships can both facilitate and act as a barrier for home modification implementation. Carpenter and Mulligan note that family caregivers may hold onto values and ethics (e.g., communication and autonomy) that differ from the resident’s views [25]. These differing values may lead to difficulty with consensus, especially as care recipients and caregivers are involved in home modification decisions [26]. Further, Chee et al. identified numerous factors that impact caregivers’ adherence to an occupational therapist’s intervention for persons with dementia [27]. Educating caregivers and family members on engaging in open conversations may elicit consensus in home modification decisions.

Additionally, participants in both focus groups discussed concerns related to finding a professional to help residents with solutions. New tools, such as mobile app directories of professionals who provide home modifications (e.g., Nguyen et al. [28]), may become useful in searching for qualified professionals. Future interventions may also focus on advocacy to change social norms that act as barriers to home modification implementation.

### 4.3. Community Level

The participants described scenarios where factors at the community level impacted the home modification process. Community resources were essential for low-income individuals to implement home modifications, providing further evidence to the assertion by McLeroy et al. that factors from each level (e.g., community and individual) of social-ecological models can be intertwined and impact one another [29]. Findings from a review conducted by Simplican et al. also highlight the importance of community and indicate the specific importance of community resources for individuals with disabilities in the context of social inclusion [30]. Additionally, a study that compared participants in an aging-in-place program versus nursing home care found that the total Medicare and Medicaid costs were $1591.61 per month lower for those in the aging-in-place group [31]. Thus, funding and awareness of community resources to support aging-in-place may also reduce broader healthcare costs.

### 4.4. Societal Level

Frustrations were expressed at the societal level regarding current laws and social norms that affect the implementation of home modifications. Specifically, participants discussed the pitfalls of ADA, which was used as a “catch-all” term as this legislation does not cover the vast majority of homes. As such, there is a need for laws regarding the accessibility of residential housing. One potential direction may be utilizing visitability principles, a design approach that ensures that anyone, regardless of their mobility level or needs, can live and visit a home [32]. The National Council of Independent Living believes that single-family or owner-occupied housing, not covered by fair housing laws or constructed with federal funds, should be “visitable” [33]. Individualized solutions must be considered when establishing laws regarding the home environment because multiple factors influence the match between an individual’s abilities and their environment [34].

### 4.5. Limitations and Strengths

As only two focus groups were conducted, the investigation did not reach data saturation. Moreover, this study used secondary data where the information was not originally collected for the purpose of this analysis, and so further inquiry is needed to examine the interactive nature of factors across levels of the social-ecological model. This study provided an opportunity to explore the perspectives of both residents and professionals involved with home modifications. By having participants from multiple age groups, the researchers were also able to explore components of active aging throughout the life course [4]. Additionally, the use of a directed content analysis allowed researchers to explore constructs related to the factors identified by a social-ecological model. As such, the model helped identify the multiple-level factors that may impact individuals’ behavior.

## 5. Conclusions

This study contributes to the understanding of the factors, beyond the individuallevel to relationship, community, and societal levels, that may influence the implementation of home modifications. The results indicate that many stakeholders, including residents, family/caregivers, therapists, builders, remodelers, and policymakers, may both positively and negatively influence the implementation of home modifications. Residents and professionals focused much of the discussion around individual factors (e.g., needs and preferences), which may be influenced by the interaction of the various levels of the social-ecological model. As such, plausible opportunities exist for targeted interventions to address the individual, relationship, community, and societal factors that may affect home modifications.

## Figures and Tables

**Table 1:** Focus groups factors informed by the Centers for Disease Control and Prevention’s (CDC, 2021) social-ecological framework by level, factors, residents’ counts, and professionals’ counts (*N* = 16).

Ecological Level	Factors	Residents’ Counts	Professionals’ Counts
Individual	Needs & Preferences	44	39
	Affordability	4	11
Relationship	Resident & Family/Caregiver	10	3
	Resident & Professional	4	5
	Professional & Professional	0	4
Community	Community Resources	11	14
	Neighbors	0	2
Societal	Laws	0	7
	Social Norms	1	10

*Note.* Frequency counts of factors within each level reflected the number of times participants indicated a factor within their response.

## Data Availability

The dataset for this study is available upon reasonable request from the first author.
